# Discovery and Optimization of Selective Inhibitors of Meprin α (Part II)

**DOI:** 10.3390/ph14030197

**Published:** 2021-02-27

**Authors:** Chao Wang, Juan Diez, Hajeung Park, Christoph Becker-Pauly, Gregg B. Fields, Timothy P. Spicer, Louis D. Scampavia, Dmitriy Minond, Thomas D. Bannister

**Affiliations:** 1 Department of Molecular Medicine, Scripps Research, Jupiter, FL 33458, USA; CWang@scripps.edu (C.W.); hajpark@scripps.edu (H.P.); spicert@scripps.edu (T.P.S.); scampl@scripps.edu (L.D.S.); 2 Department of Chemistry, Scripps Research, Jupiter, FL 33458, USA; dminond@nova.edu; 3 Rumbaugh-Goodwin Institute for Cancer Research, Nova Southeastern University, 3321 College Avenue, CCR r.605, Fort Lauderdale, FL 33314, USA; mdiezi@yahoo.com; 4 The Scripps Research Molecular Screening Center, Scripps Research, Jupiter, FL 33458, USA; cbeckerpauly@biochem.uni-kiel.de; 5 Unit for Degradomics of the Protease Web, Institute of Biochemistry, University of Kiel, Rudolf-Höber-Str.1, 24118 Kiel, Germany; fieldsg@fau.edu; 6 Department of Chemistry & Biochemistry and I-HEALTH, Florida Atlantic University, 5353 Parkside Drive, Jupiter, FL 33458, USA; 7 Dr. Kiran C. Patel College of Allopathic Medicine, Nova Southeastern University, 3301 College Avenue, Fort Lauderdale, FL 33314, USA

**Keywords:** meprin α, meprin β, zinc metalloproteinase, medicinal chemistry, probe development

## Abstract

Meprin α is a zinc metalloproteinase (metzincin) that has been implicated in multiple diseases, including fibrosis and cancers. It has proven difficult to find small molecules that are capable of selectively inhibiting meprin α, or its close relative meprin β, over numerous other metzincins which, if inhibited, would elicit unwanted effects. We recently identified possible molecular starting points for meprin α-specific inhibition through an HTS effort (see part I, preceding paper). Here, in part II, we report further efforts to optimize potency and selectivity. We hope that a hydroxamic acid meprin α inhibitor probe will help define the therapeutic potential for small molecule meprin α inhibition and spur further drug discovery efforts in the area of zinc metalloproteinase inhibition.

## 1. Introduction

Meprin α is a zinc metalloproteinase (metzincin) that has been implicated in multiple diseases, including fibrosis [1,2], and cancers [3]. Understanding meprin α’s precise role, alone or in combination with other metzincins, including its close relative meprin β, has been difficult to establish due to the lack of known selective inhibitors. While many preliminary reports for meprin inhibitors have emerged [4,5,6,7,8], no compounds with suitable potency, selectivity, and drug-like attributes for in vivo use are known. We recently reported an HTS campaign to identify lead meprin α and meprin β inhibitors [9]. We then began SAR studies to understand the pharmacophore for meprin α inhibition, to enhance potency of the best leads, and to widen or maintain their selectivity over other metzincins. These efforts led to the identification of a potential probe molecule that may allow us in the future to ascertain the therapeutic potential for small molecule meprin α inhibition in the treatment of fibrosis or cancers.

Zinc metalloproteinases are ubiquitous in human biology and their dysregulation in certain disease states has spurred many researchers to seek modulators, usually inhibitors, of their function. The primary difficulty in most drug discovery efforts is to gain selectivity for the zinc metalloproteinase of interest. Most inhibitors are characterized by having groups that tightly bind zinc ions, often through bidentate coordination. Compounds that bind strongly to zinc metalloproteinases in this way can be found, though a frequently encountered liability is poor target selectivity, since coordination to zinc, and perhaps to other metal ions of similar size, is often the main driver of potency. As a consequence, many such inhibitors block off-target zinc-and even non-zinc metalloproteinase, including enzymes that are required for a myriad of essential biological functions of healthy cells, and they thus display intolerably high toxicity.

A common strategy is to avoid metal coordinating groups altogether. A second strategy is to employ a metal coordinating group that is: (1) consistent with drug-like properties, and (2) is merely one part of an organic molecule that also has several other functional groups that contribute substantially to its binding energy for the target, and not for other targets, thus imparting an acceptable level of target selectivity. Ideally this will translate to low toxicity in in vivo use. In our efforts here, our starting point was a screening hit containing a hydroxamic acid, R–CO–NH–OH, and we sought to alter other regions of the screening hit to widen its selectivity.

**Figure 1:**
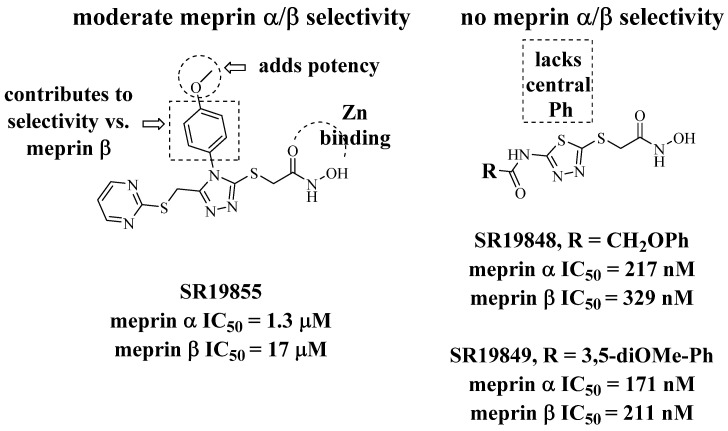
HTS hit, the aryl triazole SR19855 (left), and related, less selective thiadiazoles.

In our HTS effort (see part I), we sought any type of inhibitor, with or without metal coordinating groups. Only leads with known Zn coordinating functional groups were found to have suitable potency for follow-up, however, and any optimization effort required that we address selectivity concerns at an early stage. The hydroxamic acid compound SR19855 (Figure 1) was a preferred meprin α inhibitor hit, having both significant potency (low-micromolar inhibition of meprin α) and hints of target selectivity. In screens for selectivity, we saw 13-fold selectivity for meprin α inhibition over inhibition of the most closely-related enzyme, meprin β, and also at least 10-fold selectivity over a larger panel of metzincins, including MMP-2, MMP-3, MMP-8, MMP-9, MMP-10, MMP-14, ADAM10, and ADAM17. Given that a hydroxamic acid group can tightly bind zinc ions in all of these targets, we surmised that other regions of SR19855 were contributing to meprin α binding and to target selectivity. Further, we wished to further widen this selectivity window, with increases in potency as well, so that a probe molecule might be identified for use in order to isolate the effect of meprin α-specific inhibition in relevant biological and pharmacological contexts.

## 2. Results

### 2.1. Modeling of Lead Series

Our HTS effort and follow-up SAR-by-purchase efforts identified a number of moderately potent meprin α inhibitors bearing hydroxamic acid groups. Among these were related aryl triazoles and thiadiazoles (Figure 1). Notably, the thiadiazoles were unlike the aryl triazoles in terms of isoform selectivity: only the aryl triazoles were >10-fold selective for meprin α over meprin β. We considered isoform selectivity to be critically important, since low ligand selectivity limits the usefulness of metalloprotease inhibitors as isoform-specific probes. We surmised that the additional aromatic ring in the aryl triazoles (left) may be an important selectivity element favoring binding to meprin α over meprin β, and perhaps over other metzincins as well. In the thiadiazole series, similar compounds purchased with a thiazole core (one N replaced with CH) were much less potent, suggesting that the nitrogen atoms in the heterocyclic core are important.

**Figure 2:**
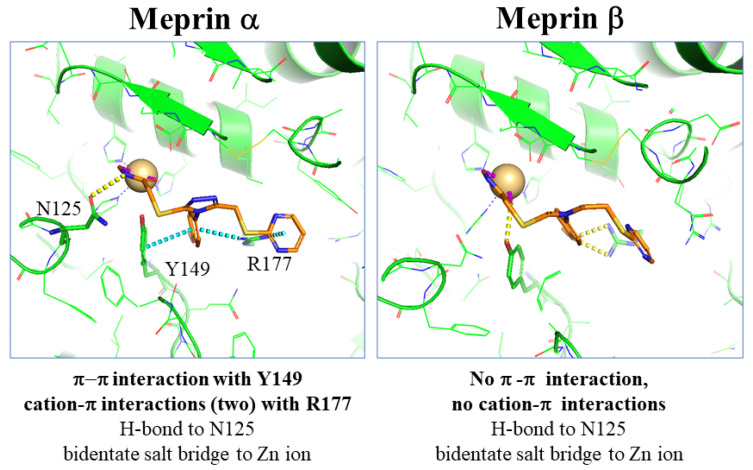
SR19855 docked to meprins α and β.

We wished to understand the possible basis for meprin α binding and SR19855 selectivity. Using coordinates for a meprin β X-ray crystal structure, we constructed a homology model for meprin α and docked several HTS hits, including the aryl triazoles. The hydroxamate of SR19885, not surprisingly, is modeled to form a strong bidentate interaction with the Zn ion in both docking models (Figure 2). This binding anchors the ligand into each active site. Overall, geometry of binding for the backbone of the ligand is similar for the meprin enzymes. Important differences are apparent for the side chains, however, especially the interactions of the central *para*-methoxy phenyl group in meprin α. As shown in the left panel of Figure 2, Y149 of meprin α makes a face-on π-π interaction with the phenyl ring. R177 residue makes cation-π interactions with both phenyl and pyrimidine rings of the ligand. Although the primary sequences of the meprin isoforms are similar in the binding pocket, these interactions are absent or are much weaker in meprin β with the exception of the hydroxamate/Zn interaction and an H-bond interaction to Y211 (Y149 in meprin α) that are preserved. These findings suggest that the aryl triazole core of the ligand should be preserved, for selectivity purposes, during our SAR studies aimed to increase potency and selectivity for meprin α.

In analyzing data from the HTS effort and for a few purchased analogs, we noted that both the phenyl and pyrimidine rings of SR19855 could not be deleted without a substantial drop in meprin α inhibitory activity. This finding agreed with our binding model (Figure 2). Together, these observations led us to explore the effect of a pyrimidine substituents and the replacement or alternative substitution of the central aryl group, with the aim of finding electronic and/or steric factors that would maximize meprin α inhibitory activity and maintain or (preferably) increase target selectivity.

### 2.2. Synthetic Strategy

**Figure 3:**
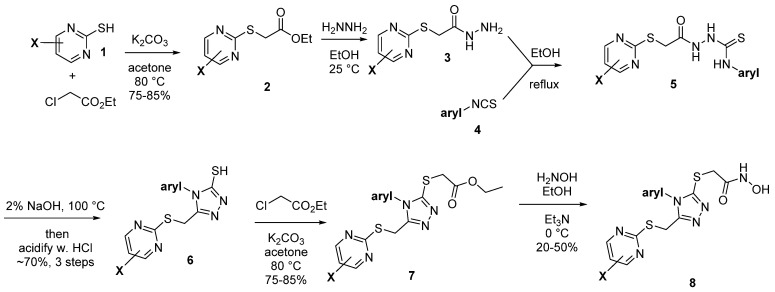
General synthesis scheme.

To further study compounds in the series, we initiated an internal synthesis effort. A versatile 6-step synthesis used is shown in Figure 3, which is adaptable for different pyrimidine X groups and for substituted phenyl rings, or a replacement heterocycle. This strategy is based upon the initial 3 steps of a literature method [10]. Briefly, the alkylation of a thiopyrimidine **1** (step 1) to give ester **2** is followed by treatment with hydrazine (step 2) to form a hydrazine amide **3**, which is acylated with an isothiocyanate **4** (step 3), which following an immediate acidic workup (step 4), gives the mercaptotriazole **6**. This potentially oxidatively sensitive intermediate is then alkylated (step 5) to give ester **7**, which can be safely stored under inert conditions and purified by chromatography, if needed. Many of the earlier intermediates are obtained in high purity through precipitation or crystallization, with minimal chromatography, as outlined in the Experimental Section. Finally, the hydroxamic acid group is installed by treatment with hydroxylamine (step 6) to give the final test hydroxamic acid product **8**.

#### 2.2.1. A. Pyrimidine Substitution

Holding the other groups constant, we varied the X substituents on the pyrimidine (Table 1), from the relatively electron-deficient unsubstituted pyrimidine, to a bulkier but neutral pyrimidine (3,5-dimethyl), to a more electron-rich 3,5-dimethoxy pyrimidine. We used symmetric disubstituted compounds due to their ready availability. Potency varied only in a small range, as all three compounds are single digit micromolar meprin α binders. Selectivity assessment using the meprin β inhibition assay, however, led us to prioritize 3,5-dimethoxy pyrimidines for further follow-up, as SR24144 showed complete lack of inhibition of meprin β at 100 μM. This contrasts with significant meprin β inhibition by SR19855 (IC_50_ = 17 μM) and modest but complete meprin β inhibition by SR24319 (IC_50_ = 60 μM). We felt that the maximization of selectivity, which was our primary criteria for probe development, offset a decrease in meprin α inhibition (meprin α IC_50_ = 8.7 μM), *provided* that meprin α inhibition could be increased by making other modifications, such as replacements for the central methoxyphenyl ring.

#### 2.2.2. Phenyl Substitution

Replacement of the central methoxyphenyl ring (Table 2) indeed allowed us to regain meprin α binding affinity without sacrificing selectivity vs. meprin β. Moving the *para* methoxy group in SR19855 to the meta or ortho position, however, reduced meprin α affinity and, more concerning, gave unwanted binding to meprin β (IC_50_ = 59 and 52 μM for SR24460 and SR24459, respectively). Reducing electron density with a pyridyl ring present or with CF_3_, Br, F, or Me groups replacing OMe maintained meprin β selectivity but had only minor effects on meprin α binding, with SR24403, with a CF_3_ group, being the best alternative (meprin α IC_50_ = 5.4 μM). A larger group, a 2-naphthyl ring system (SR24467), gave a modest increase in meprin α binding (IC_50_ = 2.0 μM) at the expense of higher molecular weight and elevated lipophilicity. We saw enhanced potency when a *para* amino substituted phenyl was used, with the 4-morpholine analog SR26466 having an IC_50_ = 1.1 μM, the 4-diethylamino analog 24,465 having an IC_50_ = 600 nM, and the 4-dimethylamino analog SR24717 being nearly equipotent, having an IC_50_ = 660 nM. IC_50_ values for each compound were determined in triplicate and the average value is given in Table 2, with SD factor as an error. Selectivity over meprin β was maintained in all analogs other than the *meta*-methoxy and *ortho*-methoxy compounds (IC_50_ vs. meprin β in all other cases was >70 μM). To test our presumption that the hydroxamic acid is essential, we also tested certain ester synthetic precursors to the hydroxamates (not shown) and saw no activity (meprin α IC_50_ > 100 μM). As shown in Table 2, SR26465, with a diethylamino group present, and SR24717, with a with a diethylamino group present, are essentially equivalent with respect to meprin α/β affinity and a selectivity ratio >100. We chose SR24417 as the lead for further evaluation.

### 2.3. SR24717 Characterization in In Vitro Assays

**Figure 4:**
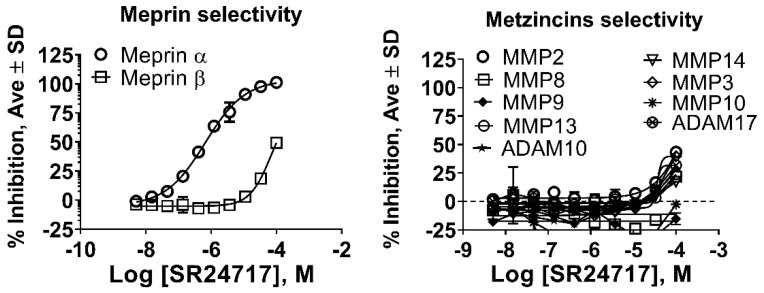
Characterization of a potential meprin α probe SR24717 against meprin β and related metzincins. (**A**) Dose response study of re-synthesis batch of SR24717 with meprin α and β; (**B**) Dose response study of re-synthesis batch of SR24717 with metzincins shows good selectivity profile. All experiments performed as 10 point 3-fold concentration-response curves in triplicate. All units are IC_50_, µM.

We tested a freshly re-synthesized, analytically pure sample of SR24717 against meprin α, meprin β, and related metzincins. IC_50_ values for SR24717 were batch independent: IC_50_ values for meprin α inhibition for multiple batches ranged from 490–670 nM. IC_50_ values for meprin β inhibition were consistently at ~70 µM or above as well. Results shown in Figure 4 are for one scale-up batch, with results in triplicate. There is full meprin α inhibition and only partial meprin β inhibition, at the higher doses tested (Figure 4A). Assay results with related metzincins demonstrated excellent broad selectivity of SR24717 (IC_50_ metzincins > 100 µM for all tested enzymes, Figure 4B).

**Figure 5:**
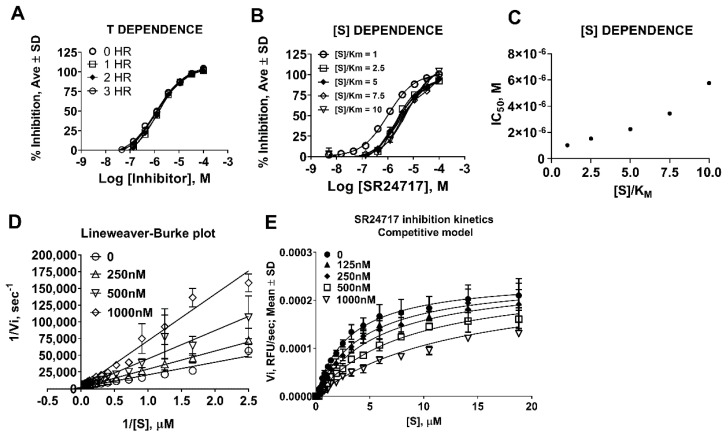
Characterization of mode of meprin α inhibition by potential probe. SR24717. (**A**) Time-dependence study of inhibition of meprin α-mediated proteolysis by SR24717. (**B**) Concentration-dependence study of meprin α inhibition-mediated proteolysis by SR24717. (**C**) Re-plot of (**B**) shows increase of IC_50_ values correlating with increase of substrate concentration suggestive of competitive inhibition mechanism by SR24717. (**D**) Lineweaver-Burke plot shows lines of best fit crossing at Y-axis, suggesting a competitive inhibition mechanism by SR24717. (**E**) Global fit of meprin α-mediated proteolysis in the presence of SR24717 to a competitive model using non-linear regression shows good fit.

We further characterized SR24717 for the mode of inhibition of meprin α. Pre-incubation of SR24717 with meprin α for 0–3 h showed no change in IC_50_ values, suggesting that SR24717 is not a time-dependent inhibitor (Figure 5A). This allowed us to use the steady-state assumption in our follow-up experiments. Varying substrate concentration in meprin α decreased the apparent potency of SR24717 (Figure 5B,C), suggesting a competitive mode of inhibition. Indeed, both linear (Figure 5D) and non-linear (Figure 5E) models showed good fit to the competitive inhibition. Global fit to the competitive model of inhibition using GraphPad Prism showed K_i_ = 300.8 nM.

**Figure 6:**
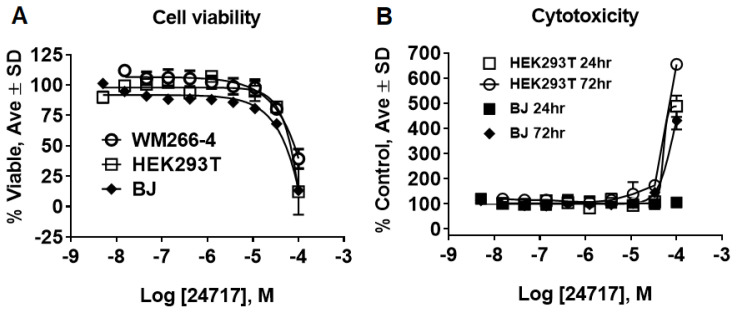
Characterization of SR24717 for effects on cell viability and cytotoxicity. (**A**) Effect of SR24717 on viability of WM266-4 (skin melanoma), HEK293 (kidney), and BJ (skin fibroblasts) cells. CellTiter Glo™ assay (Promega) was used after 72 h treatment. (**B**) Effect of SR24717 on viability of HEK293 (kidney) and BJ (skin fibroblasts) cells. The CellTox Green™ assay (Promega) was used. All experiments performed as 10-point 3-fold concentration-response curves in triplicate.

We tested SR24717 for effects on viability and cytotoxicity of several cell types. Only at a concentration of 100 µM of SR24717 were negative effects on viability and cytotoxicity observed (Figure 6), suggesting that SR24717 may be a useful probe for studying the biological role of meprin α in in vitro and in vivo systems.

## 3. Discussion

**Figure 7:**
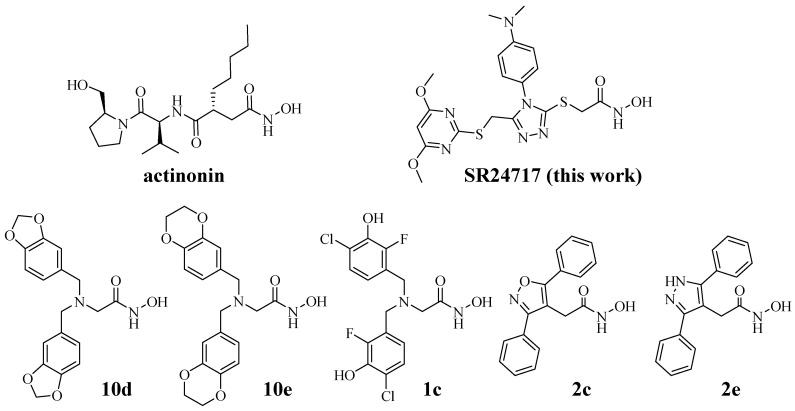
Structures of literature meprin inhibitors: actinonin, SR24717, **10d** [8]; **10e** [8], **1c** [11], **2c** [11], **2e** [11].

While we had initially hoped that HTS efforts would identify non-chelating, non-hydroxamic acid meprin α or meprin β inhibitor leads, we have demonstrated that the two aromatic groups in the lead series of meprin α inhibitors are also very important, and perhaps uniquely positioned, for meprin α binding. Thus, Zn chelation is not the overwhelming driver of ligand affinity. SR24717 has unexpectedly high selectivity vs. 10 other zinc metalloproteases (Figure 4), indicative of potential use as a probe molecule in an in vitro setting. The selectivity is noteworthy in comparison to known inhibitors (Table 3), with structures depicted in Figure 7. 

The meprin α/β fold selectivity (ratio of IC_50_ values) had been ~13 for the HTS hit SR19855 and was improved to 106 for SR24717. Actinonin is a meprin inhibitor that has long been commonly used in the literature, but its target selectivity is quite poor. It is a repurposed antibiotic agent that potently inhibits many metzincins and in cell-based environments, though it is significantly cytotoxic. Its wide use is purely historical: it was one of the first inhibitors of meprin α with some selectivity for meprin β, but off-target activity confounds the interpretations for any of actinonin’s effects, as they cannot be cleanly ascribed to meprin α inhibition. This is illustrated in Table 3, where we see sub-micromolar activity for actinonin vs. MMP-2, MMP-8, MMP-9, MMP-13, ADAM17 (and also MMP1, see footer), with low micromolar activity vs. meprin β and ADAM10. This contrasts greatly with similar data for SR24717.

At the time of initial submission of this manuscript, the most potent meprin α inhibitors known were bis-benzyl glycine hydroxamates **10d** and **10e**, both from the Ramsbeck labs [8], which displayed meprin α/β fold selectivity ratios of 18 and 19, respectively. The same group very recently gave an update on this series and related analogs [11], reporting more balanced pan tertiary amine inhibitors such as compound **1c** and also much more potent, conformationally constrained meprin α inhibitors **2c** and **2d**. Interestingly, compounds **2c** and **2d**, like SR24717, append two aromatic groups to a 5-membered ring core (Figure 7). Though selectivity IC_50_ values were not reported, most (as shown in Table 3) were weak inhibitors at 10 μM and showed more substantial inhibition at 200 μM. The most potent off-target for inhibitors **2c** and **2e** was ovastacin, with K_i_ values of 66 nM and 196 nM, respectively [11].

Our modeling study, depicted in Figure 2, suggests that two cation-π interactions are responsible for meprin α potency and selectivity. Our structure–activity relationship study supports the model, in that the 3,5-dimethoxypyrimidine and 4-dimethylaminophenyl groups of SR24717 work in concert to augment potency and selectivity. Though SR24717 is merely ~2-fold more potent than the HTS hit SR19855 in binding meprin α, it has selectivity advantages (~106-fold vs. meprin β), with consistently lower IC_50_ and/or lower maximum inhibition of nine other metzincins, when tested even at a high concentration (100 μM, see Figure 4 and Table 3).

We have developed a versatile, scalable, and operationally straightforward route to producing the SR24717 and its analogs. Future work includes characterization of all properties that are relevant for in vivo use, including the evaluation and optimization of DMPK properties, while maintaining or improving both target potency and meprin α selectivity.

## 4. Materials and Methods

### 4.1. Assay Reagents

MMP-1, MMP-2, MMP-8, MMP-9, MMP-10, MMP-13, MMP-14, ADAM10, ADAM17 and Mca-KPLGL-Dpa-AR-NH_2_ fluorogenic peptide substrate were purchased from R&D Systems (cat # 901-MP, 902-MP, 908-MP, 911-MP, 910-MP, 511-MM, 918-MP, 936-AD, 930-ADB, and ES010, respectively). All common chemicals were purchased from Sigma. NFF449 was purchased from Tocris (cat# 1391) and actinonin was from Sigma-Aldrich, St. Louis, MO, USA (cat# 01809).

### 4.2. Meprin α and Meprin β Substrate Synthesis

Meprin α and meprin β substrates (Mca-YVADAPK-(K-ε-Dnp) and Mca-EDEDED-(K-ε-Dnp), respectively) [12] were synthesized according to Fmoc solid-phase methodology on a peptide synthesizer. All peptides were synthesized as C-terminal amides to prevent diketopiperazine formation [13]. Cleavage and sidechain deprotection of peptide-resins was for at least 2 h using thioanisole-water-TFA (5:5:90). The substrates were purified and characterized by preparative RP HPLC and characterized by MALDI-TOF MS and analytical RP HPLC.

### 4.3. Meprins Expression Protocol

Recombinant human meprin α and meprin β were expressed using the Bac-to-Bac expression system (Gibco Life Technologies, Paisley, UK) as described before [5,14]. Media and supplements were obtained from Gibco Life Technologies. Recombinant Baculoviruses were amplified in adherently growing *Spodoptera frugiperda* (*Sf*)9 insect cells at 27 °C in Grace’s insect medium supplemented with 10% fetal bovine serum, 50 units/mL penicillin, and 50 μg/mL streptomycin. Protein expression was performed in 500 mL suspension cultures of BTI-TN-5B1-4 insect cells growing in Express Five SFM supplemented with 4 mM glutamine, 50 units/mL penicillin, and 50 μg/mL streptomycin in Fernbach-flasks using a Multitron orbital shaker (INFORS AG, Bottmingen, Switzerland). Cells were infected at a density of 2 × 10^6^ cells/mL with an amplified viral stock at a MOI of ~10. Protein expression was stopped after 72 h, and recombinant meprins were further purified from the media by ammonium sulfate precipitation (60% saturation) and affinity chromatography (Strep-tactin for Strep-tagged meprin α and Ni-NTA for His-tagged meprin β). Meprins were activated by trypsin, which was removed afterwards by affinity chromatography using a column containing immobilized chicken ovomucoid, a trypsin inhibitor.

### 4.4. Meprin α and Meprin β Assays in a 384-Well Plate

Both assays followed the same general protocol [15]. 5 µL of 2× enzyme solution (2.6 and 0.1 nM for meprin α and meprin β, respectively) in assay buffer (50 mM HEPES, 0.01% Brij-35, pH 7.5) were added to solid bottom black 384 low-volume plates (Nunc, cat# 264705). Next, 75 nL of test compounds or pharmacological control (actinonin) were added to corresponding wells using a 384-pin tool device (V&P Scientific, San Diego, CA, USA). After 30 min incubation at RT, the reactions were started by addition of 5 µL of 2× solutions of substrates (20 µM, meprin α Mca-YVADAPK-K(Dnp), and for meprin β Mca-EDEDED-K(Dnp)). Reactions were incubated at RT for 1 h, after which the fluorescence was measured using the Synergy H4 multimode microplate reader (Biotek Instruments) (λ_excitation_ = 324 nm, λ_emission_ = 390 nm).

Three parameters were calculated on a per-plate basis: (a) the signal-to-background ratio (S/B), (b) the coefficient for variation (CV; CV = (standard deviation/mean) × 100) for all compound test wells; and (c) the Z- or Z’-factor [16]. Z takes into account the effect of test compounds on the assay window, while Z’ is based on controls.

### 4.5. Determination of Kinetic Parameters of Meprin α Mediated Proteolysis in the Presence of Potential Probe SR24717

Substrate stock solutions were prepared at various concentrations in HTS assay buffer (50 mM HEPES, 0.01% Brij-35, pH 7.5). Assays were conducted by incubating a range of substrate (2–50 µM) and SR24717 concentrations (0–1000 nM) with 1.3 nM meprin α at 25 °C. Fluorescence was measured on a multimode microplate reader Synergy H1 (Biotek Instruments, Winooski, VT, USA) using λ_excitation_ = 324 nm and λ_emission_ = 393 nm. Rates of hydrolysis were obtained from plots of fluorescence versus time, using data points from only the linear portion of the hydrolysis curve. The slope from these plots was divided by the fluorescence change corresponding to complete hydrolysis and then multiplied by the substrate concentration to obtain rates of hydrolysis in units of μM/s. Kinetic parameters were calculated by non-linear regression analysis using the GraphPad Prism 8.0 suite of programs.

### 4.6. ADAM10 and ADAM17 Assays

Both assays followed the same general protocol. 2.5 µL of 2× enzyme solution (20 nM) in assay buffer (10 mM HEPES, 0.001% Brij-35, pH 7.5) were added to solid bottom black 384 plates (Greiner, cat# 789075). Next, test compounds and pharmacological controls were added to corresponding wells using a 384-pin tool device (V&P Scientific, San Diego). After 30 min incubation at RT, the reactions were started by addition of 2.5 µL of 2× solutions of substrate (R&D Systems cat#: ES010, Mca-KPLGL-Dpa-AR-NH_2_, 20 µM). Reactions were incubated at RT for 2 h, after which the fluorescence was measured using a Perkin Elmer Viewlux multimode microplate imager (λ_excitation_ = 324 nm, λ_emission_ = 393 nm). All compounds were tested in 10-point, 1:3 serial dilutions dose-response format starting with highest concentration of 100 µM. IC_50_ values were determined using non-linear regression analysis using the GraphPad Prism 8.0 suite of programs. 

### 4.7. MMP Assays

All assays followed the same general protocol. 5 µL of 2× enzyme solution (5 nM) in assay buffer (50 mM Tricine, 50 mM NaCl, 10 mM CaCl_2_, 0.05% Brij-35, pH 7.5) were added to solid-bottom black 384 plates (Nunc, cat# 264705). Next, test compounds and pharmacological controls were added to corresponding wells using a 384-pin tool device (V&P Scientific, San Diego). After 30 min incubation at RT, the reactions were started by addition of 5 µL of 2× solutions of MMP substrate (R&D Systems cat#: ES010, 20 µM). Reactions were incubated at RT for 1 h, after which the fluorescence was measured using the Synergy H4 multimode microplate reader (Biotek Instruments) (λ_excitation_ = 324 nm, λ_emission_ = 390 nm). All compounds were tested in 10-point, 1:3 serial dilutions dose-response format starting with highest concentration of 100 µM. IC_50_ values were determined using non-linear regression analysis using the GraphPad Prism 8.0 suite of programs.

### 4.8. Cell Toxicity Studies

Test compounds were solubilized in 100% DMSO and added to polypropylene 384-well plates (Greiner cat# 781280). 1250 of BJ skin fibroblasts or primary melanocytes were plated in 384-well plates in 8 µL of serum-free media (HybriCare for BT474, EMEM for HEK293). Test compounds and pharmacological assay control (lapatinib) were prepared as 10-point, 1:3 serial dilutions starting at 10 mM, then added to the cells using the pin tool mounted on Integra 384. Plates were incubated for 72 h at 37 °C, 5% CO_2_ and 95% RH. After incubation, 8 µL of CellTiter-Glo^®^ (Promega cat# G7570) was added to each well and incubated for 15 min at room temperature. Luminescence was recorded using a Biotek Synergy H1 multimode microplate reader. Viability was expressed as a percentage relative to wells containing media only (0%) and wells containing cells treated with DMSO only (100%). Three parameters were calculated on a per-plate basis: (a) the signal-to-background ratio (S/B), (b) the coefficient for variation (CV; CV = (standard deviation/mean) × 100) for all compound test wells, and (c) the Z’-factor. IC_50_ values were calculated by fitting normalized data to sigmoidal log vs. response equation utilizing non-linear regression analysis from GraphPad Prism 8.

### 4.9. Molecular Modeling Studies

The crystal structure of human meprin β (PDB ID 4GWN) was used as a template for constructing a model of human meprin α using Prime (Schrödinger, LLC, New York, NY, USA), with the option of a single template to build a single chain and including the Zn ion. The homology model and the coordinates, 4GWN, were prepared using the protein preparation wizard in Maestro v12.2 (Schrodinger, LLC, NY, USA). Docking studies were performed using Glide SP v8.7 (Schrodinger, LLC, NY, USA) with no constraint. The docking grid was generated around the Zn ion with a box size of 18 × 18 × 18 Å3. SR19855 was prepared for Glide docking with LigPrep (Schrodinger, LLC, NY, USA) to include different conformational states. The docking pose with the highest docking score for each compound was then merged to the docked structures for energy minimization using the OPLS3e force field (Schrodinger, LLC, NY, USA) and the results were analyzed in pymol. 

### 4.10. Compound Synthesis and Characterization

Synthesis of all test compounds followed the 6-step route summarized in Figure 3, which was adapted from the literature [10,17,18]. Details for all steps in the synthesis of SR24717 are shown, with supporting data. Other compounds followed the same synthetic methods, and data follows for all analogs reported herein.

Step 1, Ethyl 2-[(aryl)thio] acetate derivatives (**2**). Mercaptopyrimidine **1** (1 equiv.), ethyl 2-chloroacetate (2 equiv.) and potassium carbonate (1.2 equiv.) were heated at 80 °C in acetone. Reaction progress was monitored by LC/MS and after no more than 2 h, the mixture was cooled, filtered, and concentrated in vacuo to give the crude product **2**, which was dissolved in minimal dichloromethane, precipitated by addition of diethyl ether, and the precipitate was collected by filtration and dried under high vacuum. The product was isolated in 75% yield, with >95% purity by LC/MS, with no chromatography necessary.

Step 2, 2-[(Aryl)thio]acetohydrazide derivatives (**3**). A mixture of ethyl 2-[(aryl)thio]acetate (**2**) (1 equiv.) and hydrazine hydrate (2 equiv.) in ethanol was stirred for 1–2 h at room temperature. The colorless hydrazide **3** precipitated out of the solution and was collected by filtration. The precipitate was washed with water, dried under high vacuum, and was taken to the next step without purification. It was >95% pure by LC/MS analysis. 

Step 3, Thiosemicarbazide derivatives (**5**). A solution of the hydrazide (**3**) (1 equiv.) and an aryl isothiocyanate **4**, typically a substituted phenyl isothiocyanate (1 equiv.), in hot ethanol was refluxed for 1 h. The colorless thiosemicarbazide **5** precipitated out of the solution and, after cooling, it was collected by filtration. The precipitate was washed with water, ether, dried under high vacuum, and was taken to the next step without purification. It was >95% pure by LC/MS analysis. 

Step 4, Mercaptotriazole derivatives (**6**). A suspension of thiosemicarbazide (**5**) in aq. 2% NaOH was heated at 100 °C, and the solution became homogeneous. Reaction progress was monitored by LC/MS and cyclization was complete within 2 h. The solution was then cooled to room temperature. The pH was adjusted to 6–7 by the addition of 3 M HCl, at which point a colorless precipitate formed. The precipitate collected by filtration, washed with water, and dried under high vacuum. It was >95% pure by LC/MS analysis and was obtained in 70% isolated yield for steps 2–4, from compound **2**. This intermediate was potentially sensitive to air oxidation, so it was kept under inert atmosphere and then carried without delay to the next step.

Step 5, Mercaptotriazole esters (**7**). A mixture of the mercaptotriazole (**6**) (1 equiv.), ethyl 2-chloroacetate (1.12 equiv.) and potassium carbonate (1.5 equiv.) in acetone was heated at 80 °C. Reaction progress was monitored by LC/MS and the alkylation was complete within 2 h. Upon cooling, a colorless precipitate formed and it was collected by filtration. The precipitate washed with water and was dried under high vacuum. The precursor to the probe candidate was >95% pure by LC/MS analysis and was obtained in 85% isolated yield. Certain mercaptotriazole esters analogs were less crystalline and could be purified by column chromatography on silica gel at this stage, using a CH_2_Cl_2_ to 5% MeOH in CH_2_Cl_2_ gradient. Intermediate ester **7** was stable and could be stored cold under inert atmosphere for later use in the final step. 

Step 6, final hydroxamic acids (**8**). A solution of mercaptotriazole ester (**7**) (1 equiv.) in MeOH/DCM (3:1) was cooled to 0 °C and was treated with saturated hydroxylamine hydrochloride solution (6 equiv.) followed by the addition of sat. aq. NaOH (12 equiv.). After reaction completion (typically~10 min) the mixture was concentrated in vacuo, water was added, and the pH was adjusted to 7.0 by the addition of 2 M HCl. A colorless precipitate formed (25–30% yield, >95% analytical purity by HPLC) and it was collected by filtration. The product was kept cold and under inert atmosphere to prevent slow hydrolysis of the hydroxamic acid to a carboxylate, presumably from atmospheric moisture. Such hydrolysis was also seen upon prep HPLC purification, so the final product was best isolated by precipitation.

Characterization data for the potential meprin α inhibitor SR24717: ^1^H NMR (600 MHz, DMSO-*d*_6_): δ ppm 10.75 (s, 1H), 9.01 (s, 1H), 8.58 (s, 1H), 7.16 (d, *J* = 8.8 Hz, 2H), 6.68 (d, *J* = 8.8 Hz, 2H), 5.89 (s, 1H), 6.94 (s, 1H), 4.48 (s, 2H), 3.81 (s, 6H), 3.78 (s, 2H), 2.94 (s, 6H). ^13^C NMR (DMSO-*d*_6_): δ ppm 170.9, 168.2, 164.2, 153.7, 151.9, 151.2, 128.4, 120.3, 112.4, 86.0, 60.2, 33.4, 24.4. MS (ESI, M + H) calcd for (C_19_H_23_N_7_O_4_S_2_ + H): 478.13, found 477.86, purity by analytical HPLC >95%.

### 4.11. Data for Other Test Compounds

#### 4.11.1. SR19855, *N*-hydroxy-2-((4-(4-methoxyphenyl)-5-((pyrimidin-2-ylthio)methyl)-4*H*-1,2,4-triazol-3-yl)thio)acetamide

Colorless solid, 42% yield for the final step. Other steps were comparable in yield to that for SR24717. Silica gel chromatography used MeOH/DCM 0–5% gradient, on the penultimate step. ^1^H NMR (400 MHz, DMSO-*d*_6_): δ ppm 10.76 (s, 1H), 9.02 (s, 1H), 8.54 (d, *J* = 4.88 Hz, 2H), 7.37 (d, *J* = 8.92 Hz, 2H), 7.19 (t, *J* = 4.88 Hz, 1H), 7.04 (d, *J* = 8.92 Hz, 2H), 4.42 (s, 2H), 3.80 (s, 6H), 3.77 (s, 2H). ^13^C NMR (600 MHz, DMSO-*d*_6_): δ ppm 170.0, 163.7, 160.2, 157.8, 153.0, 151.0, 128.9, 124.9, 117.5, 114.9, 55.5, 33.1, 23.9. MS (ESI, M + H) calcd for (C_16_H_16_N_6_O_3_S_2_ + H): 405.07, found 405.01, purity by analytical HPLC > 95%.

#### 4.11.2. SR24139, 2-((5-(((4,6-dimethylpyrimidin-2-yl)thio)methyl)-4-(4-methoxyphenyl)-4*H*-1,2,4-triazol-3-yl)thio)-*N*-hydroxyacetamide

Colorless solid, 45% yield for final step. Other steps were comparable in yield to that for SR24717. ^1^H NMR (400 MHz, DMSO-*d*_6_): δ ppm 10.75 (d, *J* = 1.2 Hz, 1H), 9.02 (d, *J* = 1.2 Hz, 1H), 7.35 (d, *J* = 8.9 Hz, 2H), 7.00 (d, *J* = 8.9 Hz, 2H), 6.93 (s, 1H), 4.47 (s, 2H), 3.79 (s, 6H), 3.78 (s, 2H), 2.28 (s, 6H). ^13^C NMR (600 MHz, DMSO-*d*_6_): δ ppm 167.7,167.0, 163.7, 160.01, 153.3, 151.0, 128.8, 124.8, 116.2, 114.7, 55.5, 33.1, 23.5, 23.2. MS (ESI, M + H) calcd for (C_18_H_20_N_6_O_3_S_2_ + H): 433.10, found 433.05, purity by analytical HPLC > 97%.

#### 4.11.3. SR24144, 2-((5-(((4,6-dimethoxypyrimidin-2-yl)thio)methyl)-4-(4-methoxyphenyl)-4*H*-1,2,4-triazol-3-yl)thio)-*N*-hydroxyacetamide

Colorless solid, 45% yield for the final step, recrystallized from EtOAc. Other steps were comparable in yield to that for SR24717. ^1^H NMR (400 MHz, DMSO-*d*_6_): δ ppm 10.76 (d, *J* = 0.88 Hz, 1H), 9.01 (d, *J* = 0.88 Hz, 1H), 8.37 (d, *J* = 8.92 Hz, 2H), 7.01 (d, *J* = 8.92 Hz, 2H), 5.91 (s, 1H), 4.49 (s, 2H), 3.80 (s, 6H), 3.79 (s, 3H), 3.77 (s, 2H). ^13^C NMR (600 MHz, DMSO-*d*_6_): δ ppm 170.5, 167.7, 163.7, 160.2, 153.0, 151.1, 128.9, 124.8, 114.8, 85.5, 55.5, 54.3, 33.1, 24.0. MS (ESI, M + H) calcd for (C_18_H_20_N_6_O_5_S_2_ + H): 465.09, found 465.01, purity by analytical HPLC > 95%.

#### 4.11.4. SR24460, 2-((5-(((4,6-dimethoxypyrimidin-2-yl)thio)methyl)-4-(3-methoxyphenyl)-4*H*-1,2,4-triazol-3-yl)thio)-*N*-hydroxyacetamide

Colorless solid, 38% yield for the final step. Other steps were comparable in yield to that for SR24717. ^1^H NMR (400 MHz, DMSO-*d*_6_): δ ppm 10.77 (d, *J* = 1.04 Hz, 1H), 9.03 (d, *J* = 1.48 Hz, 1H), 7.42 (dt, *J* = 7.88, 0.80 Hz, 1H), 7.04 (q, *J* = 7.08 Hz, 3H), 5.91 (s, 1H), 4.55 (s, 2H), 3.80 (s, 6H), 3.75 (s, 2H). ^13^C NMR (600 MHz, DMSO-*d*_6_): δ ppm 170.5, 167.7, 163.6, 159.8, 152.7, 150.6, 133.44, 130.5, 119.3, 115.6, 113.2, 85.5, 55.5, 54.2, 33.2, 24.0. (ESI, M + H) calcd for (C_18_H_20_N_6_O_5_S_2_ + H): 465.09, found 465.02, purity by analytical HPLC > 98%.

#### 4.11.5. SR24459, 2-((5-(((4,6-dimethoxypyrimidin-2-yl)thio)methyl)-4-(2-methoxyphenyl)-4*H*-1,2,4-triazol-3-yl)thio)-*N*-hydroxyacetamide

Colorless solid, 40% yield for the final step. Other steps were comparable in yield to that for SR24717.^1^H NMR (400 MHz, DMSO-*d*_6_): δ ppm 10.77 (s, 1H), 9.03 (s, 1H), 7.50 (dt, *J* = 6.88, 1.56 Hz, 1H), 7.39 (dd, *J* = 7.8, 1.56 Hz, 1H), 7.21 (d, *J* = 8.4 Hz,1 H), 7.05 (t, *J* = 7.64 Hz, 1H), 5.91 (s, 1H), 4.48 (d, *J* = 15.0 Hz, 1H), 4.37 (d, *J* = 15.0 Hz, 1H), 3.81 (s, 6H), 3.75 (s, 2H). ^13^C NMR (600 MHz, DMSO-*d*_6_): δ ppm 170.5, 167.8, 163.7, 154.3, 153.0, 151.2, 132.0, 129.1, 120.8, 120.5, 112.8, 85.5, 55.9, 54.2, 33.2, 23.8. (ESI, M + H) calcd for (C_18_H_20_N_6_O_5_S_2_ + H): 465.09, found 464.98, purity by analytical HPLC > 95%.

#### 4.11.6. SR24718, 2-((5-(((4,6-dimethoxypyrimidin-2-yl)thio)methyl)-4-(pyridin-3-yl)-4*H*-1,2,4-triazol-3-yl)thio)-*N*-hydroxyacetamide

Colorless solid, 42% yield for the final step. Other steps were comparable in yield to that for SR24717. ^1^H NMR (400 MHz, DMSO-*d*_6_): δ ppm 10.82 (s, 1H), 9.09 (s, 1H), 8.80 (d, *J* = 2.36 Hz, 1H), 8.75 (dd, *J* = 4.8, 1.4 Hz, 1H), 8.07 (td, *J* = 8.24, 2.48 Hz, 1H), 7.62 (dd, *J* = 8.24, 4.84 Hz, 1H), 5.98 (s, 1H), 4.63 (s, 2H), 3.87 (s, 6H), 3.84 (s, 2H). ^13^C NMR (600 MHz, DMSO-*d*_6_): δ ppm 170.5, 167.5, 163.5, 152.9, 151.0, 150.9, 148.2, 135.6, 129.6, 124.4, 85.6, 54.3, 33.7, 24.0. MS (ESI, M + H) calcd for (C_16_H_17_N_7_O_4_S_2_ + H): 436.08, found 435.91, purity by analytical HPLC > 95%.

#### 4.11.7. SR24003, 2-((5-(((4,6-dimethoxypyrimidin-2-yl)thio)methyl)-4-(4-(trifluoromethyl)phenyl)-4*H*-1,2,4-triazol-3-yl)thio)-*N*-hydroxyacetamide

Colorless solid, 35% yield for final step. Other steps were comparable in yield to that for SR24717. ^1^H NMR (400 MHz, DMSO-*d*_6_): δ ppm 10.78 (d, 1H), 9.02 (d, 1H), 7.88 (d, *J* = 8.31 Hz, 2H), 7.76 (d, *J* = 8.24 Hz, 2H), 5.88 (s, 1H), 4.58 (s, 2H), 3.79 (s, 9H). ^13^C NMR (600 MHz, DMSO-*d*_6_): δ ppm 170.5, 167.5, 163.5, 152.8, 150.5, 136.1, 130.4, 128.8, 126.8, 124.5, 122.7, 85.5, 54.3, 33.6, 24.0. MS (ESI, M + H) calcd for (C_18_H_17_F_3_N_6_O_4_S_2_ + H): 503.07, found 503.01, purity by analytical HPLC > 97%.

#### 4.11.8. SR24462, 2-((4-(4-bromophenyl)-5-(((4,6-dimethoxypyrimidin-2-yl)thio)methyl)-4*H*-1,2,4-triazol-3-yl)thio)-*N*-hydroxyacetamide

Colorless solid, 35% yield for the final step. Other steps were comparable in yield to that for SR24717. ^1^H NMR (400 MHz, DMSO-*d*_6_): δ ppm 10.76 (s, 1H), 9.03 (s, 1H), 7.70 (d, *J* = 8.7 Hz, 2H), 7.45 (d, *J* = 8.7 Hz, 2H), 5.93 (s, 1H), 4.55 (s, 2H), 3.82 (s, 6H), 3.78 (s, 2H). ^13^C NMR (600 MHz DMSO-*d*_6_): δ ppm 170.5, 167.51, 163.6, 152.7, 150.6, 132.7, 131.8, 129.7, 123.6, 85.5, 54.3, 33.4, 24.0 MS (ESI, M + H) calcd for (C_17_H_17_BrN_6_O_4_S_2_ + H): 513.0, 515.0, found 512.99, 514.95, purity by analytical HPLC > 95%.

#### 4.11.9. SR24463, 2-((5-(((4,6-dimethoxypyrimidin-2-yl)thio)methyl)-4-(4-fluorophenyl)-4*H*-1,2,4-triazol-3-yl)thio)-*N*-hydroxyacetamide

Colorless solid, 35% yield for the final step. Other steps were comparable in yield to that for SR24717. ^1^H NMR (400 MHz, DMSO-*d*_6_): δ ppm 10.77 (s, 1H), 9.03 (s, 1H), 7.55–7.58 (m, 2H), 7.37 (t, *J* = 8.8 Hz, 2H), 5.93 (s, 1H), 4.53 (s, 2H), 3.82 (s, 6H), 3.79 (s, 2H). ^13^C NMR (600 MHz, DMSO-*d*_6_): δ ppm 170.5, 167.6, 163.4, 161.7, 152.8, 150.8, 130.1, 128.8, 116.8, 85.5, 54.3, 33.3, 24.0. MS (ESI, M + H) calcd for (C_17_H_17_FN_6_O_4_S_2_ + H): 453.07, found 452.59, purity by analytical HPLC > 96%.

#### 4.11.10. SR24467, 2-((5-(((4,6-dimethoxypyrimidin-2-yl)thio)methyl)-4-(m-tolyl)-4*H*-1,2,4-triazol-3-yl)thio)-*N*-hydroxyacetamide

Colorless solid, 42% yield for the final step. Other steps were comparable in yield to that for SR24717. ^1^H NMR (400 MHz, DMSO-*d*_6_): δ ppm 10.76 (s, 1H), 9.02 (s, 1H), 7.50 (t, *J* = 7.59 Hz, 1H), 7.31 (d, *J* = 7.47 Hz, 1H), 7.24 (d, *J* = 6.75 Hz, 2H), 5.90 (s, 1H), 4.51 (s, 2H), 3.80 (s, 9H), 2.29 (s, 3H). ^13^C NMR (600 MHz, DMSO-*d*_6_): δ ppm 170.5, 167.7, 163.6, 152.8, 150.7, 139.6, 132.4, 130.4, 129.5, 127.7, 124.5, 85.5, 54.3, 33.2, 24.0, 20.6. (ESI, M + H) calcd for (C_18_H_20_N_6_O_4_S_2_ + H): 449.10, found 448.94, purity by analytical HPLC > 95%.

#### 4.11.11. SR26467, 2-((5-(((4,6-dimethoxypyrimidin-2-yl)thio)methyl)-4-(naphthalen-1-yl)-4*H*-1,2,4-triazol-3-yl)thio)-*N*-hydroxyacetamide

Colorless solid, 32% yield for the final step. Other steps were comparable in yield to that for SR24717. Silica gel chromatography used MeOH/DCM 0–5% gradient, on the penultimate step. ^1^H NMR (400 MHz, DMSO-*d*_6_): δ ppm 10.76 (s, 1H), 9.02 (s, 1H), 8.06 (d, *J* = 8.32 Hz, 1H), 8.03 (d, *J* = 7.44 Hz, 1H), 7.67 (d, *J* = 7.36 Hz, 1H), 7.62–7.54 (m, 3H), 7.13 (d, *J* = 8.16 Hz, 1H), 5.74 (s, 1H), 4.41 (d, *J* = 15.1 Hz, 1H), 4.38 (d, *J* = 15.2 Hz, 1H), 3.80 (s, 2H), 3.59 (s, 6H). ^13^C NMR (600 MHz, DMSO-*d*_6_): δ ppm 170.1, 167.2, 163.6, 153.9, 151.8, 133.7, 130.9, 128.8, 128.4, 128.3, 128.1, 127.1, 127.0, 125.5, 121.4, 85.4, 54.0, 33.1, 23.7. MS (ESI, M + H) calcd for (C_21_H_20_N_6_O_4_S_2_ + H): 485.10, found 484.91, purity by analytical HPLC > 95%.

#### 4.11.12. SR26466, 2-((5-(((4,6-dimethoxypyrimidin-2-yl)thio)methyl)-4-(4-morpholinophenyl)-4*H*-1,2,4-triazol-3-yl)thio)-*N*-hydroxyacetamide

Colorless solid, 41% yield for the final step. Other steps were comparable in yield to that for SR24717. ^1^H NMR (400 MHz, DMSO-*d*_6_): δ ppm 10.76 (s, 1H), 9.02 (s, 1H), 7.24 (d, *J* = 8.96 Hz, 2H), 6.97 (d, *J* = 9.04 Hz, 2H), 5.91 (s, 1H), 4.50 (s, 2H), 3.81 (s, 6H), 3.78 (s, 2H), 3.75 (t, *J* = 4.88 Hz, 4H), 3.17 (t, *J* = 4.76 Hz, 4H). ^13^C NMR (600 MHz, DMSO-*d*_6_): δ ppm 170.5, 167.7, 163.7, 153.1, 151.6, 151.3, 128.1, 122.6, 114.7, 85.6, 66.0, 54.3, 47.4, 33.0, 24.0. MS (ESI, M + H) calcd for (C_21_H_26_N_7_O_5_S_2_ + H): 520.14, found 519.95, purity by analytical HPLC > 95%.

#### 4.11.13. SR26465, 2-((4-(4-(diethylamino)phenyl)-5-(((4,6-dimethoxypyrimidin-2-yl)thio)methyl)-4*H*-1,2,4-triazol-3-yl)thio)-*N*-hydroxyacetamide

Colorless solid, 41% yield for the final step. Other steps were comparable in yield to that for SR24717. ^1^H NMR (400 MHz, CD3CN-*d*_6_): δ ppm 7.06 (d, *J* = 8.88 Hz, 2H), 6.59 (d, *J* = 9.04 Hz, 2H), 5.71 (s, 1H), 4.51 (s, 2H), 3.80 (s, 6H), 3.69 (s, 2H), 3.33 (q, *J* = 7.04 Hz, 4H), 1.11 (t, *J* = 7.08 Hz, 6H). ^13^C NMR (600 MHz, DMSO-*d*_6_): δ ppm 170.4, 167.7, 163.8, 153.3, 151.6, 148.1, 128.2, 118.8, 111.0, 85.6, 54.2, 43.7, 32.8, 23.8, 12.3. MS (ESI, M + H) calcd for (C_21_H_27_N_7_O_4_S_2_ + H): 506.16, found 505.96, purity by anal. HPLC > 95%.

## 5. Conclusions

The lead molecule emerging from this study, SR24717, optimized from a high throughput screening hit, shows a promising profile with selectivity for meprin α. Three interactions, a π-π interaction and two cation-π interactions, appear to be responsible for this selectivity vs. meprin β and other metzincins. Accordingly, modification of substituent effects on the aryl and heteroaryl rings impact affinity and selectivity. Compounds in this chemical series may prove useful for the study of the efficacy and safety of meprin α-selective inhibition in animal models of human disease.

## Figures and Tables

**Table 1:** Pyrimidine substitution: dimethoxylation disfavors meprin β inhibition.

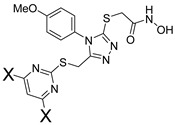	**X**	**ID**	**Meprin α IC_50_, μM**	**Meprin β IC_50_, μM**
	H	SR19855	1.3 ± 0.1	17 ± 3
	Me	SR24139	4.1 ± 0.8	60 ± 7
	OMe	SR24144	8.7 ± 1.1	>100

**Table 2:** SAR studies in the central aromatic ring.

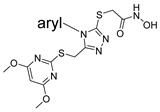			
**aryl**	**ID**	**Meprin α IC_50_, μM**	**Meprin β IC_50_, μM**
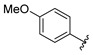	SR19855	1.3 ± 0.1	17 ± 3
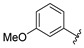	SR24460	10.3 ± 1.1	59 ± 10
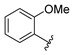	SR24459	12.1 ± 3.9	52 ± 1
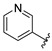	SR24718	10.1 ± 2.3	>100
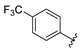	SR24003	5.4 ± 1.2	>100
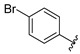	SR24462	11.2 ± 2.6	>100
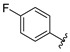	SR24463	10.4 ± 2.8	>100
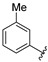	SR24467	10.3 ± 2.2	>100
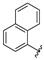	SR26467	2.0 ± 0.3	>100
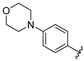	SR26466	1.1 ± 0.1	>100
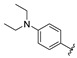	SR26465	0.60 ± 0.06	75 ± 15
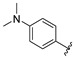	SR24717	0.66 ± 0.03	70 ± 5

**Table 3:** Selectivity testing of SR24717 and literature meprin α inhibitors.

ID	Meprin α	Meprin β	MMP2	MMP3	MMP8	MMP9	MMP10	MMP13	MMP14	ADAM10	ADAM17
SR24717 ^a^	0.66 ± 0.03	70 ± 5	>100	>100	>100	>100	>100	>100	>100	>100	>100
Actinonin ^b^	0.004	4.8 ± 0.5	0.09	NR	0.19	0.1 ± 0.01	NR	0.1	>1000	2.2 ± 0.2	0.2 ± 0.02
**10d** [8] ^c^	0.16 ± 0.001	2.95 ± 0.35	95% 26%			91% 61%		86% 55%		86% 61%	86% 57%
**10e** [8]	0.40 ± 0.03	7.59 ± 0.01	94% 65%			91% 21%		98% 47%		80% 56%	80% 41%
**1c** [11] ^d^	0.19 ± 0.001	0.03 ± 0.35	95%			91%		86%		86%	86%
**2c** [11] ^e^	0.004 ± 0.001	0.813 ± 0.001	87%			86%		80%		80%	62%
**2e** [11] ^f^	0.003 ± 0.001	0.199 ± 0.001	104%			81%		75%		73%	61%

All units are IC_50_, µM, % values indicate % activity remaining at 10 μM (top) and at 200 μM (bottom). All assays performed in triplicate, with error bars ± SD. a. Multiple synthesis of SR24717 batches were tested, shown are results of one representative batch. b. Actinonin also has IC_50_ = 0.1 μM vs. MMP1. c. Compound **10d** has IC_50_ = 1.15 μM vs. ovastacin [11]. d. Compound **1c** has IC_50_ = 0.49 μM vs. ovastacin [11]. e. Compound **2c** has K_i_ = 66 nM vs. ovastacin [11]. f. Compound **2e** has K_i_ = 196 nM vs. ovastacin [11].

## Data Availability

The data presented in this study are available on request from the corresponding author.

## Abbreviations

MMP	matrix metalloprotease
ADAM	a disintegrin and metalloprotease
